# Associations Between Implementation of the Caregiver Advise Record Enable (CARE) Act and Health Service Utilization for Older Adults with Diabetes: Retrospective Observational Study

**DOI:** 10.2196/32790

**Published:** 2022-06-21

**Authors:** Yaguang Zheng, Bonnie Anton, Juleen Rodakowski, Stefanie C Altieri Dunn, Beth Fields, Jacob C Hodges, Heidi Donovan, Connie Feiler, Grant Martsolf, Andrew Bilderback, Susan C Martin, Dan Li, Alton Everette James

**Affiliations:** 1 Meyers College of Nursing New York University New York, NY United States; 2 Wolff Center at University of Pittsburgh Medical Center Pittsburgh, PA United States; 3 Department of Occupational Therapy University of Pittsburgh Pittsburgh, PA United States; 4 Department of Kinesiology School of Education University of Wisconsin-Madison Madison, WI United States; 5 School of Nursing University of Pittsburgh Pittsburgh, PA United States; 6 Healthwise Boise, ID United States; 7 Graduate School of Public Health University of Pittsburgh Pittsburgh, PA United States

**Keywords:** electronic health record, caregiver, diabetes, hospital readmission, emergency department utilization, CARE Act, EHRs, older adults, utilization

## Abstract

**Background:**

The Caregiver Advise Record Enable (CARE) Act is a state level law that requires hospitals to identify and educate caregivers (“family members or friends”) upon discharge.

**Objective:**

This study examined the association between the implementation of the CARE Act in a Pennsylvania health system and health service utilization (ie, reducing hospital readmission, emergency department [ED] visits, and mortality) for older adults with diabetes.

**Methods:**

The key elements of the CARE Act were implemented and applied to the patients discharged to home. The data between May and October 2017 were pulled from inpatient electronic health records. Likelihood-ratio chi-square tests and multivariate logistic regression models were used for statistical analysis.

**Results:**

The sample consisted of 2591 older inpatients with diabetes with a mean age of 74.6 (SD 7.1) years. Of the 2591 patients, 46.1% (n=1194) were female, 86.9% (n=2251) were White, 97.4% (n=2523) had type 2 diabetes, and 69.5% (n=1801) identified a caregiver. Of the 1801 caregivers identified, 399 (22.2%) received discharge education and training. We compared the differences in health service utilization between pre- and postimplementation of the CARE Act; however, no significance was found. No significant differences were detected from the bivariate analyses in any outcomes between individuals who identified a caregiver and those who declined to identify a caregiver. After adjusting for risk factors (multivariate analysis), those who identified a caregiver (12.2%, 219/1801) was associated with higher rates of 30-day hospital readmission than those who declined to identify a caregiver (9.9%, 78/790; odds ratio [OR] 1.38, 95% CI 1.04-1.87; *P*=.02). Significantly lower rates were detected in 7-day readmission (*P*=.02), as well as 7-day (*P*=.03) and 30-day (*P*=.01) ED visits, among patients with diabetes whose identified caregiver received education and training than those whose identified caregiver did not receive education and training in the bivariate analyses. However, after adjusting for risk factors, no significance was found in 7-day readmission (OR 0.53, 95% CI 0.27-1.05; *P*=.07), 7-day ED visit (OR 0.63, 95% CI 0.38-1.03; *P*=.07), and 30-day ED visit (OR 0.73, 95% CI 0.52-1.02; *P*=.07). No significant associations were found for other outcomes (ie, 30-day readmission and 7-day and 30-day mortality) in both the bivariate and multivariate analyses.

**Conclusions:**

Our study found that the implementation of the CARE Act was associated with certain health service utilization. The identification of caregivers was associated with higher rates of 30-day hospital readmission in the multivariate analysis, whereas having identified caregivers who received discharge education was associated with lower rates of readmission and ED visit in the bivariate analysis.

## Introduction

Recent data from the Centers for Disease Control and Prevention have shown that 29.2% of older Americans (aged ≥65 years) have diabetes [1]. Older adults with diabetes endure many daily health management challenges surrounding their diet, blood glucose levels, medication and insulin injections, and skin and foot care [2-6]. Due to these challenges, older adults are more likely to experience acute and chronic complications related to their diabetes, which can subsequently lead to increased health care service utilization including hospitalization, emergency department (ED) visit, and mortality [7,8]. The total direct and indirect costs attributed to diabetes in the United States substantially increased from US $261 billion in 2012 to US $327 billion in 2017 [9].

Among older adults with diabetes, caregivers can play a critical role in helping older adults with diabetes maintain or improve their health [10]. A family caregiver need not be related to the patient by blood or marriage; a friend, neighbor, partner, or paid caregiver could be identified by the patient as serving in this role [11]. A caregiver can assist with tasks at home such as medication management, dietary adherence, and skin and foot care [12,13]. They can also help organize complex medication regimens, operate specialized medical equipment, and communicate with and coordinate care by multiple providers [13]. A cross-sectional study indicated that patients with diabetes who have a caregiver were more likely to report moderate or high medication adherence than those with no caregivers [14]. Another study showed that inpatient diabetes education for patients or caregivers is associated with reduced hospital readmission among patients with poor glycemic control [15]. Furthermore, a recent systematic review and meta-analysis of 11 randomized controlled trials found that the integration of caregivers into the discharge planning process significantly reduced the risk of hospital readmission compared to noninclusion of caregivers for older adult patients discharged to home [16]. However, 50% of family caregivers looking after spouses or partners do not receive sufficient assistance or training from health care professionals to complete skilled medical or nursing tasks [17]. This is problematic because a position statement from the Association of Diabetes Care & Education Specialists addressed the importance of preparing the patient and caregiver to perform self-management survival skills by the time of discharge [18]. Collectively, the evidence demonstrates the importance of including and educating caregivers to help alter the unfavorable trajectories of the outcomes for older adults with diabetes.

The Caregiver Advise Record Enable (CARE) Act [19,20] supports the inclusion and education of caregivers in hospital discharge planning. Since the law’s introduction in 2014, it has already been mandated in 40 states and territories [21] and requires hospitals to implement procedures to identify and educate caregivers [11]. Given the recent introduction of the CARE Act, we aimed to understand the impact of the implementation of the CARE Act on health service utilization outcomes (ie, hospital readmission, ED visits, and mortality) of older patients with diabetes. The following research questions were asked: (1) Were there differences in health service utilization between pre- and postimplementation of the CARE Act? (2) Was there an association between the identification of caregivers for older adult patients with diabetes and health service utilization outcomes? and (3) Was having identified caregivers who received education and training on how to care for an older patient with diabetes associated with more positive health service utilization outcomes than patients whose identified caregivers did not receive education and training?

## Methods

### Study Design and Setting

This was a retrospective, observational study. The CARE Act was implemented for inpatients at the University of Pittsburgh Medical Center (UPMC). As an integrated finance and delivery system, the UPMC presented a unique opportunity to study the implementation of the CARE Act.

### Sample

Data were retrieved from patients who were admitted as inpatients, were aged ≥65 years at time of admission (the reason for admission did not need to be a diabetes diagnosis), had an associated International Classification of Diseases 10th Revision diagnosis of diabetes (E10-E14) [22], and were discharged between May and October 2017. This time period was selected because the CARE Act was implemented at UPMC in April 2017. The data before the implementation of the CARE Act were retrieved from September 2016 to mid-March 2017. Data were excluded from the analysis for patients who received care from skilled nursing, rehabilitation, or home health care services; those who transferred to another hospital; or patients who were considered same day observations or same day surgery patients.

### Implementation of the CARE Act

The Pennsylvania CARE Act was implemented in UPMC, a large integrated academic health center. The CARE Act includes 3 main requirements [20,23-25]: (1) ask each patient if they would like to identify a caregiver at admission to hospital, (2) notify the caregivers prior to discharge about the discharge occurring, and (3) educate and train the caregiver. UPMC has specific sections in the electronic health record (EHR) system dedicated to complying with the CARE Act. First, the admission screen was designed to instruct providers to ask the patient if they wanted to identify and record the contact information (eg, relationship with the patient such as spouse, children, or partner) of caregivers. The admitted hospital inpatients are given the option to identify a caregiver to participate in their discharge education and training. Patients can choose to decline identifying a caregiver. For example, a patient with a clinical background may not feel a need to identify a caregiver, or a patient may not have anyone available for support once they are home. Second, if a caregiver is identified and recorded, the intention is for providers to coordinate the discharge planning so that the caregivers can be present. The discharge notification screen is used to notify patients that their caregivers can schedule a visit time for discharge education and training. Third, a patient or caregiver education and training screen was applied where providers could document the different types of educational content and delivery modes (eg, tube feedings, dressing changes, medication management, foot care, teach-back method). The education and training can occur over multiple sessions. Staff were encouraged to perform a “teach-back” process to verify that the caregiver understood what was taught.

### Measurement

All data were retrieved from the EHR. The independent variables included caregiver identification (yes vs no) and caregiver education (yes vs no). The dependent variables included 7-day and 30-day hospital readmissions, 7-day and 30-day ED visits, and 7-day and 30-day mortality. Outcome intervals were from the date of index discharge date. The risk factors included age, race, sex, marital status, income, the number of comorbid conditions (Elixhauser comorbidity index) [26], admission to the intensive care unit (ICU), ICU length of stay (LOS), and surgery. Other variables included insurance type, caregiver relationship to patient, and the reasons of hospitalization.

### Statistical Analysis

SPSS statistics software (version 25; IBM Corp) was used for analysis. Descriptive statistics for continuous variables, such as age, ICU LOS, and the number of comorbidities, were reported as mean (SD). Categorical variables, such as gender and marital status, were described using frequency counts and percentages. Likelihood-ratio chi-square tests (bivariate tests) were used to test whether (1) there were differences in health service utilization between pre- and postimplementation of the CARE Ac; (2) caregiver identification status (yes vs no) was individually associated with each outcome (7- or 30-day hospital readmissions, 7- or 30-day ED visits, and mortality); and (3) providing education to identified caregivers (yes vs no) was individually associated with each outcome. Subsequently, multivariate logistic regression models were applied to examine each analysis after adjusting for risk factors. A *P* value <.05 was considered statistically significant.

### Ethics Approval

The study received Quality Improvement approval (ID 634) from UPMC. It has been vetted for ethics and approved for dissemination outside the organization.

## Results

A total of 2591 patients (Table 1) with diabetes were included in our analyses. The mean age of participants was 74.6 (SD 7.1) years. Of the 2591 patients, 46.1% (n=1194) were female; 86.9% (n=2251) were White and 9.4% (n=243) were Black; 97.4% (n=2523) had type 2 diabetes; 56.7% (n=1475) were married; and the mean income was US $47,853 (SD 15,223). Clinical characteristics showed that patients had a mean Elixhauser comorbidity index of 5.0 (SD 2.0). Of these 2591 patients, 10.3% (n=286) had a stay in the ICU and the mean hospital LOS was 3.7 (SD 3.1) days. The most common reasons for hospitalization included acute kidney failure (6.5%, n=168), hypertensive heart disease with heart failure (5.8%, n=150), non-ST elevation myocardial infarction (5.1%, n=132), sepsis (2.4%, n=62), atrial fibrillation (2.1%, n=54), chronic obstructive pulmonary disease with (acute) exacerbation (2%, n=52), pneumonia (1.9%, n=49), chronic obstructive pulmonary disease with acute lower respiratory infection (1.8%, n=47), urinary tract infection (1.4%, n=36), and transient cerebral ischemic attack (1.2%, n=31). The most common payers were Medicare Part A (39.9%, n=1034), UPMC for Life Medicare Health Maintenance Organization (19.8%, n=514), Security Blue Referred (8.4%, n=218), Advantra-Medicare Health Maintenance Organization (5%, n=130), and Freedom Blue (4.2%, n=108).

**Table 1:** Differences in characteristics between caregiver identified and caregiver declined.

Characteristic			Overall (N=2591)		Caregiver identified (n=1801)		Caregiver declined (n=790)		*P* value^a^
Age (years), mean (SD)			74.6 (7.1)		74.7 (7.0)		74.3 (7.1)		.16
Elixhauser comorbidity index, mean (SD)			5.0 (2.0)		5.0 (2.0)		5.1 (2.1)		.25
LOS^b^ (days), mean (SD)			3.7 (3.1)		3.7 (3.0)		3.7 (2.9)		.99
Income (US $), mean (SD)			47,853 (15,223)		48,097 (15,150)		47,296 (15,383)		.22
ICU^c^, yes, n (%)			286 (11)		195 (10.8)		73 (9.2)		.22
Gender, female, n (%)			1194 (46.1)		812 (45.1)		382 (48.4)		.12
Marital status, married, n (%)			1475 (56.9)		1114 (61.9)		361 (45.7)		<.001
Race, White, n (%)			2251 (86.9)		1569 (87.1)		682 (86.3)		.66
Surgery, n (%)			618 (23.9)		460 (25.5)		158 (20)		.002
Diabetes, type 2, n (%)			2523 (97.4)		1748 (97.1)		775 (98.1)		.22
**Comorbidities, n (%)**									
	Hypertension	1329 (51.3)		927 (51.5)		402 (50.9)		.78	
	Heart failure	841 (32.5)		575 (31.9)		266 (33.7)		.38	
	Kidney failure	729 (28.1)		524 (29.1)		205 (25.9)		.10	
	Myocardial infarction	232 (9)		150 (8.3)		82 (10.4)		.10	
	Stroke	73 (2.8)		49 (2.7)		24 (3)		.66	
	Hyperlipidemia	31 (1.2)		25 (1.4)		6 (0.8)		.16	

^a^Compares differences between caregiver identified and caregiver declined.

^b^LOS: length of stay.

^c^ICU: intensive care unit.

We compared the differences in health service utilization between pre- and postimplementation of the CARE Act; however, no significance was found. We then compared the differences in health service utilization between those who identified a caregiver and those who declined to identify a caregiver after implementation of the CARE Act. Of the 2591 patients, 1801 (69.5%) identified a caregiver, whereas 790 (30.5%) declined to identify a caregiver. The caregiver relationship to patient (note: there missing values for this variable for 7 participants) included spouse (55%, 986/1794), child (28.7%, 516/1794), parent (0.1%, 2/1794), and other (16.2%, 290/1794). Patients who identified a caregiver were more likely to be married (*P*<.001) and hospitalized for surgery (*P*=.002) than those who declined to identify a caregiver (Table 1). No significant differences were found in any patient outcomes between individuals who identified a caregiver and those who declined to identify a caregiver in the bivariate analyses (Table 2). However, after adjusting for risk factors (including Elixhauser comorbidity index, ICU [no/yes], ICU LOS, age, medical/surgical patient type, race, sex, marital status, and median zip code income) to perform the multivariate analyses, the 30-day readmission rate among the patients who identified a caregiver (12.2%, 219/1801) was significantly higher than the rate for patients who declined to identify a caregiver (9.9%, 78/790; odds ratio [OR] 1.38, 95% CI 1.04-1.87; *P*=.02). No significant differences were found in 7-day hospital readmission,7-day and 30-day ED visits, or 7-day and 30-day mortality between the 2 groups after adjusting for the risk factors (Table 2).

**Table 2:** Differences in patient outcomes between caregiver identified and caregiver declined.

Patient outcome		Caregiver identified (n=1801), n (%)	Caregiver declined (n=790), n (%)	Unadjusted *P* value		Risk-adjusted *P* value^a^		Odds ratio (95% CI)
**Readmission**								
	7-Day	82 (4.6)	30 (3.8)	.38	.16		1.37 (0.88-2.13)	
	30-Day	219 (12.2)	78 (9.9)	.09	.02		1.38 (1.04-1.83)	
**Emergency department visit**								
	7-Day	135 (7.5)	53 (6.7)	.47	.28		1.21 (0.86-1.69)	
	30-Day	302 (16.8)	128 (16.2)	.72	.24		1.15 (0.91-1.45)	
**Mortality**								
	7-Day	11 (0.6)	3 (0.4)	.44	.36		1.91 (0.48-7.58)	
	30-Day	27 (1.5)	12 (1.5)	.97	.68		1.17 (0.56, 2.42)	

^a^Risk-adjusted variables included the number of comorbid conditions, intensive care unit (no/yes), intensive care unit length of stay, age, medical/surgical patient type, race, sex, marital status, and income.

Among the 1801 patients who identified caregivers, 399 (22.2%) caregivers received education, whereas 1402 (77.8%) did not receive education. Patients with diabetes whose identified caregiver received education were more likely to be surgical patients (*P*=.01), male (*P*<.001), and married (*P*<.001) than those whose identified caregiver did not receive education (Table 3). In the bivariate analyses, significantly lower rates of 7-day readmission (*P*=.02) as well as 7-day (*P*=.03) and 30-day (*P*=.01) ED visits were detected among patients with diabetes whose identified caregiver received education than those whose identified caregiver did not receive education. After risk adjustment (multivariate analyses), there was no significant decrease in 7-day readmission (OR 0.53, 95% CI 0.27-1.05; *P*=.07) and 7-day (OR 0.63, 95% CI 0.38-1.03; *P*=.07) and 30-day (OR 0.73, 95% CI 0.52-1.02; *P*=.07) ED visits. No significant associations were found for the other outcomes before or after adjusting for risk factors (Table 4).

**Table 3:** Differences in characteristics between identified caregivers who received education and training and those who did not receive education and training.

Characteristic			No education and training, (n=1402)		Received education and training, (n=399)		*P* value
Age (years), mean (SD)			74.7 (7.0)		74.8 (7.0)		.70
Elixhauser comorbidity index, mean (SD)			5.0 (2.0)		4.99 (2.0)		.77
LOS^a^ (days), mean (SD)			3.7 (3.0)		3.7 (3.1)		.94
Income (US $), mean (SD)			47,687 (14,689)		49,542 (16,612)		.03
ICU^b^, yes, n (%)			147 (10.5)		48 (12)		.39
Gender, female, n (%)			672 (47.9)		140 (35.1)		<.001
Marital status, married, n (%)			810 (57.8)		304 (76.2)		<.001
Race, White, n (%)			1213 (86.5)		356 (89.2)		.005
Surgery, n (%)			338 (24.1)		122 (30.6)		.01
Diabetes, type 2, n (%)			1359 (96.9)		389 (97.5)		.43
**Comorbidities, n (%)**							
	Hypertension	722(51.5)		205 (51.4)		.97	
	Heart failure	457 (32.6)		118 (29.6)		.25	
	Kidney failure	410 (29.2)		114 (28.6)		.79	
	Myocardial infarction	113 (8.1)		37 (9.3)		.44	
	Stroke	37 (2.6)		12 (3)		.69	
	Hyperlipidemia	16 (1.1)		9 (2.3)		.11	

^a^LOS: length of stay.

^b^ICU: intensive care unit.

**Table 4:** Differences in patient outcomes between identified caregivers who received education and training and those who did not receive education and training.

Patient outcome		Received education and training (n=399), n (%)		No education and training, (n=1402), n (%)		Unadjusted *P* value		Risk-adjusted *P* value^a^		Odds ratio (95% CI)
**Readmission**										
	7-Day	10 (2.5)	72 (5.1)		.02		.07		0.53 (0.27-1.05)	
	30-Day	39 (9.8)	180 (12.8)		.09		.14		0.76 (0.52-1.10)	
**Emergency department visit**										
	7-Day	20 (5.0)	115 (8.2)		.03		.07		0.63 (0.38-1.03)	
	30-Day	51 (12.8)	251 (17.9)		.01		.07		0.73 (0.52-1.02)	
**Mortality**										
	7-Day	1 (0.3)	10 (0.7)		.25		.26		0.30 (0.04-2.42)	
	30-Day	4 (1.0)	23 (1.6)		.33		.32		0.57 (0.19-1.71)	

^a^Risk-adjusted variables included the number of comorbid conditions, intensive care unit (no/yes), intensive care unit length of stay, age, medical/surgical patient type, race, sex, marital status, and income.

## Discussion

Our primary focus was to examine the relationships among the CARE Act identification and education tenets and the various health service utilization outcomes of older adults with diabetes. We found that the identification of a caregiver for older patients with diabetes being discharged to home was significantly associated with higher 30-day readmission rates after adjusting for risk factors. We also found that having a caregiver who received education was associated with lower rates of 7-day readmission and 7-day and 30-day ED visits, but these associations were not significant after adjusting for risk factors.

Our findings suggest that patients with diabetes who identified a caregiver were at an increased risk for 30-day hospital readmission. One possibility is that the identification of a caregiver is simply a proxy for more serious illnesses or higher care needs following surgery. This finding aligns with a descriptive qualitative study that showed that patients with diabetes who require a caregiver are at higher risk for hospital readmission [27]. In addition, a retrospective, case-control study using deidentified EHR data found that 10% of patients with diabetes were readmitted within 30 days of discharge [28], which is similar with the 30-day readmission rate in patients who declined a caregiver (9.9%) but lower than that in those who identified a caregiver in our study (12.2%). Although our study did not explore factors that may have contributed to the increased 30-day hospital readmission rate in patients who identified a caregiver, several potential reasons may exist (eg, patients having more complex medical conditions [29], a severe issue that needs further care after hospitalization [29,30], or an escalation of diabetic treatments such as insulin injections [28,30]). It also might be attributed to caregivers paying more attention to the patients’ abnormal signs or symptoms. We believe further investigation and study of these factors is warranted.

We also found that having a caregiver who received education was associated with lower rates of 7-day readmission and 7-day and 30-day ED visits than those whose identified caregiver did not receive education, but these association did not remain significant after adjusting for risk factors. This finding might indicate that educating caregivers to properly care for older patients with diabetes is important for reducing health service utilization, as these patients are at a high risk of acute and chronic complications related to their illness [31]. In addition, caregivers might reduce the burden of the many daily tasks associated with relevant diabetes management [2,3,32].

Our study did not find associations between identifying a caregiver or caregiver education with the mortality rate for older patients with diabetes. This might be attributed to the low incidence of mortality, small sample size, comorbidities, or the time frame selected for data analyses. To our knowledge, no published articles have evaluated the impact of CARE Act implementation on mortality rate. Therefore, future work could investigate this effect using a longer duration since only 6 months of data were retrieved.

The study has several limitations. The sample consisted of mostly White patients with a high mean income, limiting any conclusions related to racially and ethnically diverse samples. Additionally, the study was not designed to assess causality or identify a mechanism by which improvement occurs. Other potential confounding factors not included in the analysis (eg, severity of disease and clinical documentation issues) may have influenced the patients’ outcomes. Moreover, the design does not truly account for the policy implementation, and as a result it is not clear if other hospital policies or practices in place at the time that could have influenced these results. Another limitation of the study is the short time range of the EHR data. It would be beneficial to determine if these findings could be replicated using a larger sample size over a longer period of time. Furthermore, although all the patients had diabetes, they were not necessarily in the hospital for a reason related to diabetes. The patient’s utilization of health services outside of UPMC was also not captured. However, this would be present for both groups—those who identified a caregiver and those who did not.

In conclusion, our study found that the implementation of the CARE Act was associated with certain health service utilization changes. The identification of caregivers was associated with higher rates of 30-day hospital readmission in the multivariate analysis, whereas the identification of caregivers who received education was associated with lower rates of readmission and ED visit in the bivariate analysis. Future research directions are aimed at determining whether patient outcomes are influenced by the education delivered (who, what, when, and how) to hospitalized patients with diabetes and their caregivers [32].

## Abbreviations

CARE Act: Caregiver Advise Record Enable Act
ED: emergency department
EHR: electronic health record
ICU: intensive care unit
LOS: length of stay
OR: odds ratio
UPMC: University of Pittsburgh Medical Center
